# Editorial: Adolescent storm and stress: a 21st century evaluation

**DOI:** 10.3389/fpsyg.2023.1257641

**Published:** 2023-08-03

**Authors:** Christy M. Buchanan, Daniel Romer, Laura Wray-Lake, Sheretta T. Butler-Barnes

**Affiliations:** ^1^Department of Psychology, Wake Forest University, Winston-Salem, NC, United States; ^2^Annenberg Public Policy Center, Annenberg School for Communication, University of Pennsylvania, Philadelphia, PA, United States; ^3^Luskin School of Public Affairs, University of California, Los Angeles, Los Angeles, CA, United States; ^4^Brown School of Social Work, Washington University in St. Louis, St. Louis, MO, United States

**Keywords:** adolescence, storm and stress, risk-taking, emotions, parent-child conflict, wellbeing, positive development

## Introduction

Adolescence, a period covering ~10–18 years of age, has long been characterized as a time of “storm and stress”. The characterization entered psychology from research on adolescent development done by Hall (1904). Although Hall's view is now recognized as extreme, his characterization has influenced popular opinion, research, policy, and practice relevant to adolescents ever since, despite reasons to believe that such a characterization is not accurate or helpful. Arnett (1999) described three primary domains of a storm and stress characterization: risk behavior, mood disruption, and parent-child conflict, arguing that negative (i.e., undesirable, unsafe, challenging) behavior in each domain during adolescence poses challenges for youth and the adults around them.

In this Research Topic, we solicited scholarly analysis evaluating the premises and the impact of the “storm and stress” characterization. We aimed, through these analyses, to articulate a conceptualization of adolescents in the 21st century that adequately considers the diversity of developmental experience that currently exists. To promote this aim, we examine the articles in this Research Topic with respect to the premises and impact of a “storm and stress” characterization. We organize our analysis around three relevant themes: (a) the typicality of negative and positive behaviors during adolescence; (b) predictors of “typical” adolescent behavior; and (c) the impact of storm and stress characterizations on adolescents. We conclude by articulating a characterization of adolescence for the 21st Century that is more consistent with the data than is a “storm and stress” characterization. Although bolstered by the findings reported in this Research Topic, our suggested characterization is not altogether new, so we also reflect on what is needed to establish this more accurate characterization of adolescents among scholars, professionals who work with adolescence, and the public.

## The “typicality” of negative and positive behaviors

A primary aspect of the storm and stress characterization of adolescence is that adolescents exhibit more negative,^1^ or undesirable, characteristics in the domains of externalizing (e.g., dangerous risk behavior), internalizing (e.g., mood disruptions, anxiety, sadness), and parent-child relationships (e.g., conflict) than do younger and older individuals; in other words, such behaviors are elevated during this time in development (Hall, 1904; Arnett, 1999; Buchanan and Bruton, 2016). Additionally, articulations of a storm and stress characterization often suggest, implicitly if not explicitly, that the level of such negative behaviors during adolescence is high in an absolute or objective sense. Although the characterization does not specify percentages of adolescents who are expected to display specific negative behaviors, Hall (1904) spoke of behavior such as “cruelty, laziness, lying and thievery” as normative (Vol. I, p. 334–335), and even today both scholarly and popular language often implies that negative behavior is common or typical (Busso et al., 2018; Hunt, 2020). A storm and stress characterization does not speak directly to positive, or more desirable, behaviors (e.g., kindness, empathy, responsibility), but a consequence of the emphasis on negative behaviors is the implication that positive behaviors are less common than negative behaviors, and perhaps low in an absolute sense or low relative to younger and older individuals.

The typicality of negative (and relative absence of positive) behaviors has been questioned in the past (Hollenstein and Lougheed, 2013; Buchanan and Bruton, 2016). Several articles in this Research Topic provide additional challenges to this aspect of a storm and stress characterization. For example, Buchanan et al. examined externalizing, internalizing, and wellbeing across the transition to adolescents in 11 cultural groups across eight countries and document more contrary than supporting evidence. Although externalizing problems increased and wellbeing decreased as children moved into and across adolescence in several countries, internalizing most often peaked in childhood. Consistent with the findings that internalizing often decreased between childhood and adolescence, Di Guinta et al. found that Colombian and Italian adolescents' self-efficacy about regulating negative emotions increased (for anger) or plateaued (for sadness) over adolescence, rather than decreased, as a storm and stress perspective would predict. Perhaps more importantly, across both of these studies, negative behaviors and emotions were not the norm, and positive indicators (e.g., emotional efficacy, wellbeing) were high on average, in all cultural groups studied.

Substance use is a specific negative risk-taking behavior typically associated with adolescence, negative because of the health and safety risks as well as the fact that it is illegal for most adolescents in many countries. Willis et al. studied substance use (alcohol, cigarettes, cannabis, and drugs) among Australian adolescents. Similar to findings about externalizing in the Buchanan et al. study, risk-taking as defined by engaging in illegal substance use increased over the adolescent period, but was atypical in the sense that it never characterized more than about one-third of adolescents. Initiation of alcohol and nicotine did not exceed 50% of adolescents until age 17, in a country where the legal age for both is 18 years.

Khurana et al. studied cigarette smoking and found that although some young adolescents are drawn to risky behavior such as cigarette smoking due to its affective appeal, their interest in continuing with the behavior wanes over time, potentially as their experience using cigarettes does not continue to be as appealing as originally thought. In this study, a minority of adolescents were drawn to such behavior; the vast majority of adolescents expected that smoking would make them feel badly, and carried high risk. Among the minority who found cigarette smoking initially appealing, the appeal declined with age. The findings are consistent with a model recognizing that adolescents might experiment with novel behavior that seems appealing, but lose interest in behaviors that carry negative health or safety consequences as they gain experience with them. The image of the impulsive and emotionally reactive adolescent, in contrast, does not fit well with these data.

Evidence for a decline in wellbeing from childhood to adolescence, and over adolescence, in the Buchanan et al. study might be interpreted as a sign of adolescent storm and stress. However, here again, patterns observed within adolescence have to be considered in a larger context, which includes that the average level of wellbeing is high. In this case, the context of the entire lifespan is also important. In both the U.S. and other countries, life satisfaction starts to decline in adolescence and continues a downward trend well into midlife (Blanchflower and Oswald, 2008; Jebb et al., 2020; Romer and Hansen, 2021). The interpretation of this trend is unclear, with some positing that it reflects the search for meaning in life, which is not always a happy journey (Steger et al., 2009). From this perspective, the decline in wellbeing across cultures during adolescence could be the beginning of a normative process reflecting the growing need to find a purpose in life rather than a reflection of unhealthy storm and stress unique to adolescence.

Increases in unhealthy or dangerous risky behavior during adolescence are concerning, particularly for the minority of adolescents who engage in such behavior frequently; similarly, declines in wellbeing might be felt acutely for some adolescents. However, an accurate characterization of adolescence should account for the reality that typical levels of dangerous risk-taking are low, typical levels of wellbeing are high, and at least with respect to wellbeing, adolescence might represent the beginning of a developmental trend that continues into adulthood. The set of findings in this Research Topic regarding the typicality of negative and positive behavior in adolescence supports previous literature and underscores the importance of interpreting evidence and other news about adolescence through a holistic and nuanced lens. For example, a plethora of recent news stories decry that today's adolescents are experiencing a mental health crisis (e.g., Moniuszko, 2023), a crisis that might have been exacerbated by, yet preceded, the COVID-19 pandemic. It is important to understand the societal and ecological factors that lead to these historical trends. It is also important to acknowledge that significant majorities of adolescents report positive moods as indicated by low (and often decreasing) levels of internalizing and high levels of wellbeing. As we will see later, correcting one-sided characterizations of young people could be important in promoting even more positive and less negative behavior among adolescents.

## Predictors of “typical” adolescent behavior

According to a storm and stress characterization, the difficulties of adolescence are caused, in large part, by universal biological developments (e.g., developmentally typical changes in hormones or the brain). A related postulate is that adolescent behavior is discontinuous from that of childhood and constitutes something new that emerges (for all or most adolescents) as a result of these developmentally new biological influences. The role of context and experiences (e.g., parenting, schools, neighborhoods, race/ethnicity, culture) receives less emphasis than biology from a storm and stress perspective. In contrast, according to ecological developmental theories, environmental forces should predict variability in behavior and wellbeing, both relative to younger and older individuals and in an absolute sense; substantial variability in levels and trajectories of behavior would contradict storm and stress assumptions of typicality.

Although no papers in this Research Topic addressed biological influences, several of them speak powerfully to the role of context, and specific experiences, in shaping adolescent behavior. For example, differences by country in levels, trajectories, and predictors of both negative and positive characteristics suggest cultural influences (Buchanan et al.; Di Guinta et al.). Buchanan et al. note that trajectories of behavior were most consistent with a storm and stress characterization (i.e., difficulties increased with entry into and transition across adolescence) in Western countries, in which storm and stress theory was initially developed and has been most widely studied (Thalmayer et al., 2021). Among U.S. adolescents, behavior varied by race and ethnicity, with European American adolescents more likely to report behavior consistent with a storm and stress characterization than African American or Hispanic adolescents (Buchanan et al.). Among Australian adolescents, Willis et al. found that substance use levels and predictors were different in rural vs. urban contexts. These findings suggest that rurality is a context that needs more attention in our efforts to accurately characterize adolescent development.

Data also challenge the storm and stress characterization of adolescent behavior as discontinuous with childhood. Willis et al. found that higher levels of childhood externalizing behavior strongly predicted adolescent externalizing behaviors. Similarly, Di Guinta et al. reported childhood predictors of adolescent emotion regulation. Higher pre-adolescent (age 10) externalizing behaviors predicted lower levels of early adolescent anger regulation self-efficacy in both Colombia and Italy; lower levels of anger regulation self-efficacy predicted more internalizing and externalizing behaviors in late adolescence. Higher levels of anger in early adolescence also predicted smaller increases in anger regulation self-efficacy over the course of adolescence. Higher pre-adolescent internalizing behaviors predicted lower sadness regulation self-efficacy in early adolescence for Italian adolescents, and levels of sadness regulation self-efficacy were then stable across adolescence. Altogether, these studies emphasize a common theme in research on predictors of adolescent behavior that raises questions about the validity of a storm and stress characterization: although negative behaviors might increase compared to childhood, they do not typically arise out of the blue. Children who exhibit more negative behavior in childhood are, on average, more likely to exhibit and experience negative behavior during adolescence and increases in such behavior over adolescence (e.g., Colder et al., 2013; Weeks et al., 2016; Elam et al., 2017; Savell et al., 2022; see Willis et al. for an exception regarding internalizing in rural contexts).

In general, findings on the importance of childhood behavior and wellbeing to adolescent wellbeing cast doubt on specific biological changes of adolescence as a primary or sole cause of increases in negative behavior. Although it is possible that unobservable biological changes beginning in childhood could have some effect, the importance of childhood behavior speaks to the possible importance of genetic influences not related to adolescent development, *per se*, to contextual influences prior to adolescence, and to developmental cascades and interactions involving individual and biological propensities in both the degree to which adolescents exhibit difficulties and the trajectory of those difficulties over the adolescent years (Romer et al., 2017; Abrams, 2022).

The studies in this Research Topic also speak to the importance of understanding the impact of lived experiences of adolescents, in that they document interactions between demographic, individual, and contextual factors. In Di Guinta et al.'s words, “... the cultural differences that emerged in the present study support the view that multiple mechanisms (cultural, familial, individual) work together in supporting or obstructing healthy personality and socio-emotional development during adolescence” (p. 14). Nonetheless, the dominant cultural characterization of adolescence often loses sight of this complexity, and a new characterization needs to bring this complexity to the fore. While acknowledging limits to the scope of influences any individual study can test, an accurate theoretical context for adolescent development should, foundationally, assume such complexity. As also noted by Ballard et al., it would be fruitful for more research to examine the impact of intersectional identities and contexts, including oppression related to race, ethnicity, gender, social class, disability, and sexual orientation, on differential experiences of storm and stress assumptions and on obstacles to positive trajectories during adolescence.

An accurate understanding of biological and contextual predictors of adolescent behavior is critical because of the implications for prevention and intervention. Assumptions that adolescent behavior is determined by biology more so than context, and by forces that are discontinuous with childhood, might lead to a sense of helplessness to prevent or intervene effectively in negative adolescent behaviors. There is some support for this hypothesis in that parents' efficacy for influencing their children declines at adolescence, at least in countries where storm and stress notions about adolescence are prevalent (Ballenski and Cook, 1982)^2^ and the decline is greater with stronger parental endorsement of storm and stress notions of adolescence (Glatz and Buchanan, 2015). Alternatively, assumptions that adolescent behavior is determined by context more so than biology could lead to greater efforts—by parents and by institutions—to positively influence adolescent behavior. A robust contrast to the storm and stress conceptualization of adolescence thus requires attention to the contexts and individual factors that can, together, support adolescents' optimal development (Lerner et al., 2021).

Two studies in the Research Topic bring attention to what such efforts might look like, including provision of opportunities to enact maturity (Ballard et al.) and to engage in healthy exploration and identity development (Defoe et al.). In their conceptual paper, Ballard et al. point out that adults' assumptions of storm and stress in adolescence lead to the creation of environments and structures for adolescents that are mismatched with their developmental needs. As a result, adolescents do not have sufficient opportunities to enact maturity. Enacting maturity includes independence in making choices across contexts; communication including debate and difficult conversations with adults and others; responsibility for self, family, and others; and leadership in school and community. Ballard et al. offer a roadmap for creating and investing in opportunities to equitably support youth with different backgrounds and identities in enacting maturity. One strategy is cultivating adult allies who use their power to amplify youth voices and create spaces where youth are welcomed, valued, and heard, and a second strategy involves redesigning youth organizations to be responsive to youth's needs to enact maturity through programming that prioritizes youth voice and leadership. These ideas align with a growing body of research documenting approaches to support youth in building power to shape their own contexts in ways that are safe and equitable (e.g., Ozer et al., 2022).

Defoe et al. write about a motivation to explore in examining how adolescents view their own motivations for engaging in substance use. They find that adolescents hardly view their own behavior as acting out of storm and stress. Instead, adolescents are more likely to indicate identity motives such as trying something new or looking cool. Exploration is often a positive and normative part of adolescent development. The authors contrast such motivations with the dominant stereotype of adolescents as risk takers that assumes that risk itself—or risk in the service of rebelling against authority—is the motive. This insight, paired with the recommendations from Ballard et al., can prompt the cultivation of opportunities for adolescent exploration and identity development that also support enacting maturity and thriving.

## The impact of a storm and stress characterization on adolescents

As with other stereotypes, the negative stereotypes that emerge from a storm and stress characterization of adolescence have the potential to create self-fulfilling prophecies (Madon et al., 2008; Buchanan and Hughes, 2009; Qu et al., 2020). This process can presumably occur through the impact of negative stereotypes and expectations on the messages, opportunities, and experiences provided to adolescents, which can ultimately lead to adolescents' internalization of negative expectations and stereotypes. Articles in this Research Topic further our understanding of when and how such self-fulfilling prophecies might occur.

Defoe et al. discuss labeling theory as a way of understanding how adolescents might internalize negative labels such as “risk-taker” or “delinquent”. Especially during a period of identity exploration and uncertainty, individuals might be especially vulnerable to adopting such labels. The authors point to existing data suggesting that “identity” can mediate the association between “labels” and behavior, although more research addressing this possibility is needed.

Ballard et al.'s analysis points to the ways in which negative expectations arising from a storm and stress characterization might create missed opportunities for developing maturity. Lack of inclination or intention to provide adolescents with meaningful and healthy autonomy-building opportunities might occur even more for some youth than others, given stigmatization of individuals based on race, gender, and sexuality, among other attributes. Similarly, susceptibility to the internalization process described above might differ for youth depending on their backgrounds (Defoe et al.).

Previous work on self-fulfilling prophecies for adolescents has focused on the beliefs and expectations of individuals (e.g., mothers, adolescents). Qu et al.'s data confirm the importance of an individual's own stereotypes (in this case, positive stereotypes concerning family obligation and school engagement) on attitudes and school engagement, and also draw our attention to the processes by which stereotypes against adolescents that exist at a group level can also be important. In this study, stereotypes at the classroom level (i.e., averaged over all students in the classroom) predicted individuals' school adjustment, above and beyond stereotypes held by the individual adolescent.

The articles in our Research Topic add to existing concerns and questions about a storm and stress characterization of adolescence and to our understanding of the processes by which this characterization can affect individual adolescents negatively. The studies point to many interesting avenues for future research, including research that could address how neighborhood, school, or even country-level stereotypes might affect adolescent behavior, even when adults close to an adolescent (e.g., parents) attempt to counter the stereotype. Because people often consider storm and stress as characteristic of adolescents as a group, even while making exceptions for individual adolescents whom they know (Arnett, 1999; Aubrun and Grady, 2000), it is important to acknowledge that stereotypes at the group or community level might promote storm and stress characteristics even among adolescents who do not hold strong stereotypes themselves.

## Conceptualizing and communicating data on adolescence

A storm and stress characterization has had a stranglehold on views of adolescence over the past century despite plenty of evidence attesting to its limitations and multiple analyses directly questioning it (Rutter et al., 1976; Dasen, 2000; Nichols and Good, 2004; Hollenstein and Lougheed, 2013; Buchanan and Bruton, 2016). Despite the weight of evidence against it, a storm and stress characterization is still widely referenced, often casually, in describing adolescence. Negative views about adolescents as a group constitute ageism, and adolescents report frequent experiences of ageism from adults in their daily lives (Huynh et al., 2016). Adults show large-scale resistance to the idea that all youth are capable of thriving and are not inherently problem-prone, and these ageist views have an insidious impact on policy and practices to promote youth thriving (Yeager et al., 2018), and on adolescent wellbeing. The articles in this Research Topic add to existing literature raising serious concerns about both the accuracy and the impact of a storm and stress characterization. What will it take to release the century-long stranglehold of a storm and stress characterization and replace it with a new, more accurate, widely held characterization of adolescence in the 21st century?

We believe it will take at least three components. *First*, we need a new vocabulary or slogan to describe adolescence, one that is as simple as “storm and stress” but that evokes positive possibilities rather than negative dangers. Laudable efforts toward the goal of promoting a more positive characterization in the past have, we argue, been thwarted by more complicated verbiage (e.g., Hollenstein and Lougheed, 2013), or a clear articulation of what does *not* describe adolescence (i.e., the lack of support for storm and stress) without an equally clear description of what *does* describe adolescence (e.g., Buchanan and Bruton, 2016). We argue that “storm and stress” has been a powerful descriptor in part because of its brevity and vividness; the alternative should be similar in these characteristics. The vocabulary should be more than neutral; it should convey a clear positive potential. For example, “experimentation” is an apt word describing adolescence that can be interpreted positively, yet given existing stereotypes of adolescence, it is likely to be interpreted negatively. Furthermore, the words should point to something unique about adolescence; for example, “growth” or “maturation” are apt descriptors for adolescence but are broadly applied to describe many periods in development.

Some candidates for vocabulary that meets these requirements that have appeared in previous work include “exploration,” “opportunity,” and “promise” (Busso et al., 2018; National Academies of Sciences, Engineering, and Medicine (NASEM), 2019; UCLA's Center for the Developing Adolescent, 2023); additional terms might include “discovery”, or “potential”. Drawing on these possibilities, instead of “storm and stress”, we propose that adolescence be characterized as a time of “promise and possibility,” “openness and opportunity,” or “exploration and discovery.” Each of these options is brief, catchy, and uses words that cover the positive potential, while leaving room for challenges and missteps. We propose that scholars of adolescence move forward with such a slogan, making a collective effort to reshape thinking about adolescence in the powerful way Hall's earlier description did.

*Second*, scholars who research and write on the challenges of adolescence, including negative behaviors, must carefully put challenges and problems into larger contexts. Challenges occur in adolescence as they do at every period of development, and it is true that certain challenges (risk-taking, mood disruptions, and parent-child conflict; Arnett, 1999) are higher in adolescence than they are before. These problems—during adolescence and at any point in the lifespan—are important to public health, and deserve attention and understanding. Negative behaviors should not, however, define adolescence. It is incumbent on researchers to carefully conduct research and articulate findings in a manner that accurately characterizes the adolescent period, and does not create or reproduce distorted negative beliefs and biased assumptions about adolescents. Toward this end, researchers are urged to consistently report absolute levels of, not just age differences or group differences in, the behaviors they study. They are urged to carefully put the challenges and problems they address into the larger context of what we know about adolescents' behaviors, the ecological and cultural context and the context of the entire lifespan, a context that shows this developmental period to be one of “promise and possibility” more so than “storm and stress”. Disciplinary organizations (e.g., Society for Research on Adolescence, Society for Adolescent Health and Medicine) supporting scholars studying adolescence can be enlisted to encourage these practices, especially in the publications over which they have editorial responsibility.

*Third*, the new slogan and accompanying information should be the subject of a marketing and media campaign that reaches beyond researchers to other professionals, parents, adolescents, and the general public. Terrific work of this type is taking place by organizations such as the Center for Parent-Teen Communication (https://parentandteen.com/about-us/), whose mission is to “help parents raise teens prepared to thrive,” and the FrameWorks Institute (https://www.frameworksinstitute.org/about/), whose mission is to “help mission-driven organizations communicate about social issues in ways that build public will to support progressive change”. FrameWorks Institute has assisted organizations such as UCLA's Center for the Developing Adolescent (2023) to craft compelling descriptions of adolescence that reframe this developmental period in a way that we believe is more widely needed. This description reads in part:

During adolescence, we are rapidly learning and adapting in ways that naturally take advantage of supportive relationships, environments, and experiences that promote positive growth and development. This makes adolescence a key window for learning and discovery as well as an opportunity to mitigate the effects of earlier adversity. Experiences that provide autonomy and choice as we explore are particularly important during these years…

…Adults working to support youth must transform dysfunctional and discriminatory systems to ensure that ALL adolescents have the support to explore, discover, and become a force for good in our communities and society.

Organizations such as the Center for Parent-Teen Communication and the FrameWorks Institute can be identified and enlisted to help change society's characterization of adolescence.

Similarly, media (including social media) outlets must be enlisted for this task and educated so as to provide better education and guidance to parents, teachers, coaches, adolescents, and the general public. There are still too many references to brain development suggesting that the adolescent brain is less than ready for self-regulation, and that confuse exploration and identity development with impulsivity and rash behavior. The news media play an incredibly powerful role in establishing and perpetuating views of adolescents (Gilliam and Bales, 2001). As we have argued, such stereotypes do adolescents no favors and ignore the challenges that they face in coping with cultural and other environmental conditions. To be successful, a campaign to educate the media would have to be explicit and wide-ranging, and the leaders for such a campaign would likely have to span researchers, practitioners, and organizations that care for adolescents and promote their development; it would take time. However, without change in the “public square” conversation, it will be difficult to create true cultural change.

Our aim in this Research Topic was to articulate a conceptualization of adolescents in the 21st century that adequately considers the diversity of developmental experience that currently exists. Based on the articles in the Research Topic and previous work, we believe that a more accurate characterization would convey more positive potential and nuance than a storm and stress characterization conveys. Given that the first quarter of this century is almost over, and a storm and stress characterization persists despite previous efforts to shift views on adolescents (Busso et al., 2018; National Academies of Sciences, Engineering, and Medicine (NASEM), 2019; UCLA's Center for the Developing Adolescent, 2023), we urge action now in the following areas: adoption of a catchy phrase to replace “storm and stress”, such as “possibility and promise”; commitment from researchers to explicitly place adolescence into the larger context of adolescent development and avoid singular and stereotyped conclusions about adolescence, especially when studying the challenges and problems of this period; and an explicit effort by organizations supporting adolescents and research on adolescents to create more marketing and media campaigns promoting this time as one of possibility and promise.

## Author contributions

CB: Conceptualization, Writing—original draft, Writing—review and editing. DR: Conceptualization, Writing—review and editing. LW-L: Conceptualization, Writing—review and editing. SB-B: Conceptualization, Writing—review and editing.

## Footnotes

^1^For ease of communication in this article, we use the term “negative” to characterize those behaviors emphasized as typical from a storm and stress characterization (i.e., risk-taking, mood disruptions, parent-child conflict). We recognize that categorizing behaviors as “negative” or “positive” oversimplifies adolescents' motivations as well as the impact or importance of the behaviors.

^2^Buchanan, C. M., Glatz, T., Selçuk, S., Skinner, A. T., Lansford, J. E., Al-Hassan, S. M., et al. (2023). Developmental trajectories of parenting self-efficacy across the transition to adolescence in nine countries and twelve cultural groups: Latent growth curve analyses (Unpublished manuscript). Department of Psychology, Wake Forest University, Winston-Salem, NC, United States.
